# Investigating Anthrax-Associated Virulence Genes among Archival and Contemporary *Bacillus cereus* Group Genomes

**DOI:** 10.3390/pathogens13100884

**Published:** 2024-10-10

**Authors:** Susanna J. Sabin, Cari A. Beesley, Chung K. Marston, Taylor K. Paisie, Christopher A. Gulvik, Gregory A. Sprenger, Jay E. Gee, Rita M. Traxler, Melissa E. Bell, John R. McQuiston, Zachary P. Weiner

**Affiliations:** 1 Laboratory Leadership Service Fellow Assigned to the National Center for Emerging and Zoonotic Infectious Diseases, CDC, Atlanta, GA 30329, USA; 2 Centers for Disease Control and Prevention, National Center for Emerging and Zoonotic Infectious Diseases, Division of High-Consequence Pathogens and Pathology, Bacterial Special Pathogens Branch, 1600 Clifton Rd, Atlanta, GA 30329, USA; 3 Oak Ridge Institute for Science and Education, Oak Ridge, TN 37830, USA

**Keywords:** anthrax, *Bacillus anthracis*, *Bacillus cereus* group, genomics, pseudogenes, gene decay

## Abstract

*Bacillus anthracis* causes anthrax through virulence factors encoded on two plasmids. However, non-*B. anthracis* organisms within the closely related, environmentally ubiquitous *Bacillus cereus* group (BCG) may cause an anthrax-like disease in humans through the partial adoption of anthrax-associated virulence genes, challenging the definition of anthrax disease. To elucidate these phenomena and their evolutionary past, we performed whole-genome sequencing on non-*anthracis* BCG isolates, including 93 archival (1967–2003) and 5 contemporary isolates (2019–2023). We produced annotated genomic assemblies and performed a pan-genome analysis to identify evidence of virulence gene homology and virulence gene acquisition by linear inheritance or horizontal gene transfer. At least one anthrax-associated virulence gene was annotated in ten isolates. Most homologous sequences in archival isolates showed evidence of pseudogenization and subsequent gene loss. The presence or absence of accessory genes, including anthrax-associated virulence genes, aligned with the phylogenetic structure of the BCG core genome. These findings support the hypothesis that anthrax-associated virulence genes were inherited from a common ancestor in the BCG and were retained or lost across different lineages, and contribute to a growing body of work informing public health strategies related to anthrax surveillance and identification.

## 1. Introduction

*Bacillus anthracis* is the causative agent of anthrax, an acute disease in humans and non-human mammals. It is a globally distributed soil-dwelling bacterium that can persist in the environment for long periods through sporulation. Anthrax in humans is contracted via four primary exposure routes. Cutaneous and gastrointestinal anthrax results from contact with or the consumption of contaminated animal products and are the most common forms. The mortality rate without treatment can reach 30% for cutaneous anthrax and 74% for gastrointestinal anthrax without treatment [1]. Injection anthrax, linked to intravenous drug use, can lead to systemic disease, with its overall mortality rate reaching 25% or exceeding 90% if meningitis develops [1,2]. Inhalation anthrax has typically been associated with the processing or handling of hides or hair from contaminated animals, with a mortality rate close to 72% [1]. This is also the mode of transmission historically associated with the use of *B. anthracis* as a bioweapon, as demonstrated during the 2001 intentional distribution of *B. anthracis* spores through the U.S. Postal Service. Of the 22 resulting infections, 11 were inhalation anthrax, which resulted in five deaths [1,3,4]. It is thus classified by the United States Departments of Agriculture and Health and Human Services as a Tier 1 select agent due to its potential to pose a severe threat to animal and human health [5].

The virulence of *B. anthracis* is largely due to two large plasmids, pXO1 and pXO2, which encode the genes for a tripartite toxin and antiphagocytic capsule. The pXO1 replicon contains genes encoding for the lethal factor (*lef*), the edema factor (*cya*), and the protective antigen (*pagA*), while pXO2 contains the genes which code for the characteristic poly-y-D-glutamic acid (PGA) capsule (*capBCADE*). Molecular tests for *B. anthracis* generally rely on the detection of these virulence factors, and PCR methods amplify small segments of DNA found on pXO1, pXO2, and the *B. anthracis* chromosome [6,7].

Taxonomically, *B. anthracis* is within a group of closely related, Gram-positive, low-GC-content, sporulating bacteria referred to here as the *Bacillus cereus* group (BCG) (also known as *Bacillus cereus sensu lato*) [8,9]. Bacteria within this group, such as *B. cereus sensu stricto* and *B. thuringiensis*, tend to be environmentally ubiquitous and opportunistically cause disease in humans. *B. cereus* is typically associated with diarrheal and emetic food poisoning, with variation in virulence across different strains, though it has emerged as a source of hospital-acquired infections [10]. *B. thuringiensis* is most known as an insect pathogen and used as a bioinsecticide [8]. However, in the genomics era, numerous other species have been introduced to the BCG and, simultaneously, the lines between BCG species have been increasingly blurred.

Close genomic relatedness and phenotypic variability within the BCG have challenged traditional taxonomic classifications, including delineations between *B. anthracis* and the rest of the BCG [11,12]. BCG taxonomy was further complicated by the discovery of new anthrax-causing bacteria that killed wild great apes in the Côte d’Ivoire and Cameroon [13,14]. *Bacillus cereus* biovar *anthracis* differed from traditional *B. anthracis* in phenotype (motility and gamma phage susceptibility) and had chromosomal DNA more like *B. cereus* isolates [15,16]. However, it harbored two virulence plasmids, pBCXO1 and pBCXO2, that showed high similarity to the pXO1 and pXO2 of *B. anthracis* [16,17]. Additional isolates, identified as *B. cereus* with *B. anthracis* toxin genes, were identified in metalworkers in Louisiana and Texas suffering from severe pneumonia, referred to as welder’s anthrax [18,19,20,21,22,23]. These isolates expressed the pXO1 toxin virulence genes but expressed different capsules and lacked pXO2 homologs [18,24]. One additional cutaneous case caused by a similar atypical *Bacillus* bacterium [25] was not associated with metalworking or a laboratory-acquired infection [26]. As emerging data continuously challenge the lines between species in the BCG and the definition of anthrax disease, it is of critical importance in clinical and public health contexts to correctly classify these organisms and understand their potential pathogenicity [1,27,28,29,30]. Also important is understanding how and when anthrax-associated virulence genes may occur in different BCG lineages.

Here, we present whole-genome sequencing data from 93 non-*anthracis* BCG isolates archived at the Centers for Disease Control and Prevention in Atlanta (CDC), Georgia, USA (1967–2003) and 5 contemporary (2019–2023) non-*anthracis* BCG isolates from clinical cases sent to the CDC. The archival strains represent bacteria characterized as *B. cereus* or *B. thuringiensis* through traditional microbiological techniques isolated from clinical or environmental contexts not associated with food poisoning or food contamination, while the contemporary strains were referred to the Zoonoses and Select Agent Laboratory at the CDC for *B. anthracis* rule-out and identification. We leveraged this unique, time-structured dataset to investigate the occurrence of anthrax-associated virulence genes in the BCG and whether the genes were introduced to non-*anthracis* bacteria by horizontal gene transfer or linear inheritance through multiple methods of genomic inference. These findings contribute to our understanding of the evolution of anthrax-associated virulence in the BCG.

## 2. Methods

### 2.1. Origin and Handling of Archival Isolates

Since the 1960s, the Centers for Disease Control and Prevention has received clinical specimens and bacterial isolates from healthcare providers, state and local public health laboratories, universities, and governmental researchers. “Genus cards” for submitted isolates identified as *Bacillus cereus* and *Bacillus thuringiensis*, which recorded an isolate identification number, standardized biochemical findings, and the source of the isolate, were reviewed. Isolates not indicated as deriving from a food source or clinical source suggesting food poisoning were selected for inclusion in the archival isolate set for whole-genome sequencing. The isolates were stored at −70 °C as blood stocks.

### 2.2. Origin and Handling of Contemporary Isolates

The contemporary isolates (2019–2023) included in this project were submitted to the Centers for Disease Control and Prevention for *Bacillus anthracis* rule-out following the receipt of a positive result for 1 out of 3 targets in *B. anthracis* real-time PCR [6]. The contemporary isolates were stored at −70 °C as spore suspensions in deionized water containing 25% glycerol.

### 2.3. DNA Preparation

Subcultures of the archival isolate stocks were incubated overnight on trypticase soy agar with 5% sheep blood at 35 °C. Using a 10 µL loop, one to three loopfuls of cells were collected from each fresh plate and used to inoculate a 2 mL, screw-top tube of ~350 µL of 0.1 mm Zirconia/Silica beads and 650 µL Tris-EDTA (0.01 M, pH 8.0) buffer. Each inoculum was vortexed horizontally at maximum speed for 2 min to perform cell lysis by bead beating. The tubes were then centrifuged at 10,000× *g* for 30 s Genomic DNA was extracted from the bead-beaten supernatant using the automated Promega Maxwell CSC instrument and Cultured Cells DNA kit (Promega, Madison, WI, USA). Following DNA extraction, the eluate was filter-inactivated using centrifugation (8000× *g* for 2 min) in Millipore Ultrafree 0.1 µM filter tubes (Millipore Sigma, Burlington, MA, USA) [31]. The filtered genomic DNA extract was then stored at −20 °C for real-time PCR (rtPCR) and genome sequencing.

### 2.4. Real-Time PCR

An rtPCR assay for *B. anthracis* was performed for all isolates, as reported elsewhere [6]. A set of three primer pairs and probes was used, targeting pXO1, pXO2, and the *B. anthracis* chromosome, which are intended to produce amplicons approximately 98, 137, and 96 base pairs in length, respectively. We used the PerfeCTa Multiplex qPCR SuperMix with Low Rox (QuantaBio, Beverly, MA, USA) and performed rtPCR on the filtered DNA extracts using an ABI 7500 Fast Dx (Applied BioSystems, Waltham, MA, USA) instrument with 40 amplification cycles. A plasmid containing all three gene targets was used as the positive control, and molecular-grade water was used for the negative, non-template controls.

### 2.5. Illumina Library Preparation and Sequencing

A subset of 39 extracts was prepared and sequenced at the CDC. A filtered genomic DNA extract was quantified using a Qubit (Invitrogen, Carlsbad, CA, USA) fluorometer, and ~400 ng of DNA per isolate was prepared for sequencing using the Illumina DNA Prep kit (Illumina, San Diego, CA, USA) per the manufacturer’s instructions. The libraries were dual-indexed using the Illumina CD Indexes kit (Illumina, San Diego, CA, USA). Amplified, purified libraries were quantified on a Qubit (Invitrogen, Carlsbad, CA USA). The contemporary isolates were sequenced on the iSeq 100 platform with iSeq 100 v2 reagent kits (150-cycles, paired-end) (Illumina, San Diego, CA, USA). Thirty-four of the archival isolates were sequenced on the MiSeq platform (Illumina, San Diego, CA, USA). Of those, 9 were sequenced using 300-cycle kits, and 25 were sequenced using 600-cycle kits. For the MiSeq, equimolar pools of 6–10 libraries were combined per sequencing run.

A subset of 59 filtered archival genomic DNA extracts were sent to the Translational Genomics Research Institute (TGen, Phoenix, AZ, USA) for sequencing. DNA was quantified using a Qubit (Invitrogen, Carlsbad, CA, USA) and each extract was normalized to 100 ng using the Integra ASSIST PLUS pipetting robot (Integra Biosciences Corp., Hudson, NH, USA). Libraries were prepared with Watchmaker’s DNA Library Prep Kit with Fragmentation (Watchmaker Genomics, Boulder, CO, USA) according to the manufacturer’s instructions with slight modifications to the resuspension volumes (42 µL post-ligation and 32 µL post-amplification). Truncated, Illumina-compatible adapters were used at a concentration of 15 µM (Integrated DNA Technologies, Coralville, IA, USA), and unique dual-index primers were added from a single-use plate (Integrated DNA Technologies. Coralville, IA, USA). Amplified libraries were quantified using a Qubit (Invitrogen, Carlsbad, CA, USA) and quality was assessed using a D5000 ScreenTape on a TapeStation 4200 System (Agilent Technologies, Santa Clara, CA, USA). The libraries were assigned to pools based on approximate genome sizes and automated equimolar pooling of the final libraries occurred on the Integra ASSIST PLUS Platform. The pools were quantified with a Qubit (Invitrogen, Carlsbad, CA, USA) and fragment size was determined with a D5000 ScreenTape (Agilent Technologies, Santa Clara, CA, USA) regional analysis. Sequencing was performed on a NovaSeq X Plus system (Illumina, San Diego, CA, USA) using the NovaSeq X Series 10B Reagent Kit (300 cycles, paired-end) following standard Illumina protocols.

### 2.6. Genome Assembly

The following processing steps were applied to paired sequencing data from all isolates, contemporary and archival, through the nf-core [32] wf-paired-end-illumina-assembly workflow (v. 2.3.0) [33] written in Nextflow (domain specific language [DSL2] v. 22.04.3, Seqera, Barcelona, Spain) [34]. Raw sequencing reads were scrubbed of human DNA and broken read pairs were discarded [35,36,37]. The remaining reads were then downsampled to approximately 100× coverage to mitigate any inadvertent amplification of sequencing errors. Genome size, read count, and original sequencing depth were calculated using KMC (v. 3.2.2) [38] and Seqtk (v. 1.4-r122) [39]. Read subsampling was completed using Seqtk (v. 1.3-r106) [39]. Any remaining PhiX sequences were removed using a BBDuk (v. 38.94) [37] adapter and quality trimming was completed using Trimmomatic (v. 0.39) [40], and overlapping read pairs were merged using FLASH (v. 1.2.11) [41]. The cleaned reads were assembled de novo using SPAdes [42], and the resulting contigs were filtered and corrected using Biopython (v. 1.68, python v 2.7.18) [43], BWA (v. 0.7.17-r1188) [44], SAMtools (v. 1.9) [45], and Pilon (v. 1.23) [46]. QUAST (v. 5.0.2) [47] and CheckM2 (v. 1.0.1) [48] were used to generate assembly metrics. Depth of assembly coverage was calculated using BEDTools (v. 2.29.2) [49]. MLST genotyping was performed on each assembly using mlst (v. 2.23.0) [50,51] and assemblies were annotated using Prokka (v. 1.14.5) [52]. Sequences of 16S rRNA were extracted from each assembly using Biopython (v. 1.68, python v 2.7.18) [43] and Barrnap (v. 0.8) [53]. The extracted 16S rRNA gene sequences underwent naïve Bayesian classification using RDP Classifier [54] for genus-level detection. The 16S rRNA gene sequences were also aligned against a database of 16S gene type strain sequences (Bioprojects PRJNA33175 and PRJNA33317) using BLASTn+ (v. 2.15.0+) and the best bitscore alignment was selected [55]. In addition, cleaned reads were classified using both Kraken (v. 1.1.1) [56] and Kraken2 (v. 2.1.3) [57] to assess the sequences for potential contamination.

### 2.7. Whole-Genome Comparison with Bacillus cereus Group Type Strains

The isolate genome assemblies were compared with *Bacillus cereus* group (BCG) type strains spanning the diversity of the BCG (Supplementary Table S1) [58,59,60,61,62,63,64,65,66,67,68,69,70,71,72,73,74,75,76,77,78].

Bidirectional average nucleotide identity (ANIb) was conducted using the wf-ani workflow [79], written in Nextflow (domain specific language [DSL2] v. 22.04.3, Seqera, Barcelona, Spain) [34], and whole-genome digital DNA:DNA hybridization (dDDH) was performed on the Type (Strain) Genome Server (TYGS) [80]. Isolates which TYGS called as potential new species due to low dDDH values with any available type strain were classified according to the closest available type strain by the dDDH *d_4_* metric and ANIb. The only discordance between TYGS identification and ANIb occurred for isolates with the highest similarity to the *B. thuringiensis* type strain. In these cases, TYGS identified the isolates as belonging to *B. cereus*, as *B. thuringiensis* could be interpreted as a sub-species within the *B. cereus sensu stricto* (*s.s.*) genomospecies clade [81]. The isolates were annotated as *B. cereus/thuringiensis*, in contrast to those of highest similarity to the *B. cereus* type strain, which were only annotated as *B. cereus.* The closest type strains and dDDH/ANIb statistics for each isolate are listed in Supplementary Table S2.

### 2.8. Annotation

Prokka (v. 1.14.5) [52] was used to annotate the genome assemblies separately from the assembly pipeline to include a custom database of translated virulence and regulator genes known to occur in virulent *B. anthracis* and the welder’s anthrax isolate G9241 in addition to the ‘Bacteria’ database (see Supplementary Table S3). Prokka was run with an e-value threshold of 1 × 10^−8^. The resulting GenBank files were screened for genes of interest in each isolate. The genes of interest coding for virulence factor proteins typically found in *B. anthracis* were *pagA* (protein sequence AJI08141.1, protective antigen), *lef* (protein sequence AJI08174.1, lethal factor), *cya* (protein sequence AJI08175.1, edema factor), *capA* (protein sequence WP_001253155.1, capsule polyglutamate synthetase), *capB* (protein sequence WP_003159943.1, capsule biosynthesis protein CapB), *capC* (protein sequence WP_000468007.1, capsular polyglutamate amide ligase/translocase PgsC), *capD* (protein sequence WP_071735531.1, capsule biosynthesis protein CapD), *capE* (protein sequence AAT28992.2, capsule biosynthesis protein CapE), *inhA* (protein sequencesYP_017301 for *inhA1* and YP_017909 for *inhA2*, M6-family metalloprotease immune inhibitors InhA1 and InhA2), and *atxA* (protein sequences AJI08122.1 for *atxA1* and AJI07941.1 for *atxA2*, anthrax toxin expression trans-acting positive regulators AtxA1 and AtxA2). We also investigated the presence of genes found in welder’s anthrax isolate G9241 thought to contribute to its virulence. These genes were the following: *certhrax* (protein sequence EAL15945.1, lethal factor precursor); *bpsX* (protein sequence EAL15985.1, transcriptional regulator, 2C LytR family); *bpsA* (protein sequence EAL15984.1, chain length determinant protein); *bpsB* (protein sequence EAL15983.1, exopolysaccharide biosynthesis protein); *bpsC* (protein sequence EAL15982.1, UTP-glucose-1-phosphate uridylyltransferase); *bpsD* (protein sequence EAL15981.1, glycosyl transferase, 2C putative); *bpsE* (protein sequence EAL15980.1, sialic acid synthase, 2C putative); *bspF* (protein sequence EAL15979.1, UDP-N-acetylglycosamine 2-epimerase, 2C putative); *bpsG* (protein sequence EAL15978.1, CMP-sialic acid synthetase, 2C putative); *hasA* (protein sequence EAL12809.1, glycosyl transferase, 2C group 2 family protein domain protein); *hasB* (protein sequence EAL12810.1, UTP-glucose-1-phosphate uridylytransferase); and *hasC* (protein sequence EAL12811.1, UDP-glucose/GDP-mannose dehydrogenase family, 2C NAD binding domain family). Lastly, we included virulence genes seen in more virulent strains of *B. cereus*, including *nheABC* (protein sequences YP_018530, YP_018531, WP_001172020; non-hemolytic enterotoxin), *cytK* (protein sequence NP_830896, cytotoxin K), and *hblABC* (protein sequences NP_832844, NP_832845, and NP_832847l; hemolysin BL binding component precursor, Hemolysin BL lytic component L2).

### 2.9. Chromosome and Plasmid Alignments

To assess whether gene annotations corresponding to a known plasmid (e.g., pXO1, pXO2) were indicative of the presence of the entire plasmid replicon, we aligned the sequencing data to reference sequences for each plasmid of interest. The reference sequences included pXO1 from the Ames Ancestor *B. anthracis* (NC_007322.2), pBCXO1 from the welder’s anthrax isolate G9241 (NZ_CP009592.1), pBCXO1 from the welder’s anthrax isolate 033B87 (NZ_CP009940.1), pXO2 from the Ames Ancestor *B. anthracis* (NC_007323.3), and pBC218 from G9241 (NZ_CP009591.1). Cleaned sequence reads were aligned to the reference sequences using bowtie2 (v. 2.5.1) [82], and the alignments were sorted with samtools (v. 1.18) [45]. VCF files were generated from the alignments using bcftools (v. 1.10.2) [83]. The depth of coverage and nucleotide identity were plotted across the reference sequence using matplotlib (v. 3.5.3) [84] in Python (v. 3.9.13).

### 2.10. Pan-Genome Characterization

Prior to using the pan-genome tool, we generated a Mash-distance-based tree of the isolate and type strain genome assemblies (see “Type strain similarity assessment” above) using Mashtree (v. 1.4.6) [85]. A group of type strains were clustered separately from any of the archival or contemporary isolates in the Mash distance tree and consisted of the following: *B. clarus*, *B. cytotoxicus*, *B. gaemokensis*, *B. bingmayonensis*, *B. arachidis*, *B. pseudomycoides*, *B. manliponensis*, and *B. rhizoplanae*. They were excluded from further analyses in addition to one archival isolate, B2797, which was found to belong to the genus *Lysinibacillus*.

The GFF files generated by Prokka were used as the input for Roary (v. 3.11.2) [86], a pan-genome characterization tool. Roary was run using the -e and n non-default options to generate a multiple-FASTA core genome alignment using MAFFT [87].

### 2.11. Gene Annotation Inspection

Gene annotations were interrogated using the gene_presence_absence.csv output from Roary to explore protein clusters and cluster quality. Protein clusters were based on a minimum of 95% identity and a Markov cluster algorithm inflation value of 1.5 [86]. Additionally, amino acid reference sequences for each gene of interest were queried against the genome assemblies using tBLASTn [55] to provide amino acid identity metrics between the annotations and reference sequences. When required, annotated sequences were aligned with the gene reference and visually inspected in MEGA-X [88].

### 2.12. Core Genome Maximum Likelihood Phylogeny

Uniform sites were removed from the core genome alignment using SNP sites (v. 2.3.3) [89], and the resulting alignment was used to generate a maximum likelihood (ML) tree using RAxML-ng (v. 1.0.1-master) [90]. On the SNP (variable) sites, we used the general time-reversible gamma substitution model with the “Lewis” model of ascertainment bias control (‘GTR + G + ASC_LEWIS’). The resulting binary file was used as the input for ML tree inference with bootstrapping (‘bs-metric fmb, tbe’).

### 2.13. Comparison of ML and Gene Presence/Absence Tree

The optimal ML phylogeny from the core genome and the accessory gene presence/absence tree was visualized and explored in FigTree (v. 1.4.4) [91] and compared with a tanglegram. The tanglegram was generated in R using the *ape* (v. 5.7-1) [92] and *Dendextend* (v. 1.17.1) [93] packages. Both trees were rooted with the *B. paramycoides* type strain. The correlation between the dendrograms was evaluated with two parameters, as described previously [94]: Baker’s Gamma association index [95] and cophenetic distance [96].

### 2.14. Clock-like Signal Test for BCG

The core genome ML phylogeny was supplied to TempEst (v. 1.5.3) [97] to determine whether the phylogeny had a clock-like signal. Each isolate was annotated with the year of isolation. The type strains were annotated with the year of isolation as noted on the associated NCBI BioSample entry or inferred from their BioSample registration dates or primary publications (see Supplementary Table S1). The “Best-fitting root” option was selected to optimize the tree.

### 2.15. Clock-like Signal Test in Genomospecies Partitions

In addition to testing for a clock-like signal in the full BCG ML phylogeny, we tested for a clock-like signal within a selection of BCG genomospecies that were well represented by our data. We constructed the core genome maximum likelihood (ML) phylogeny for the *B. cereus*, *sensu stricto* (*s.s.*) and *B. mosaicus* genomospecies and analyzed them using TempEst (v. 1.5.3) [97]. Each isolate was annotated with its year of collection, and the “Best-fitting root” option was selected to optimize the phylogeny. Type strains were excluded from this analysis.

## 3. Results

### 3.1. The Sequenced Isolates Represented Numerous Species/Subgroups within the Bacillus cereus Group

**Figure 1:**
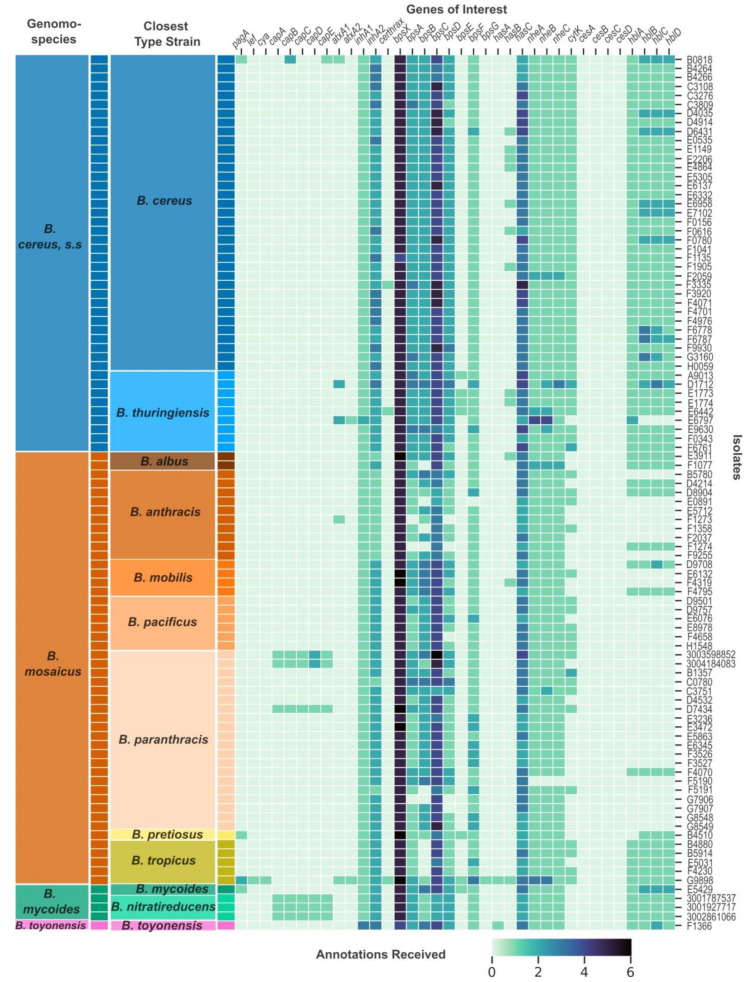
Presence and number of annotations received by virulence genes across the *Bacillus cereus* group. Each row represents a unique isolate genome, and each column represents a virulence-related gene from the *Bacillus cereus* group (BCG). The darkness of each cell represents the number of annotations that an isolate’s genomic assembly received for the corresponding gene from Prokka. The two columns on the left side of the heatmap represent the closest species type strain to that isolate as determined by ANI and dDDH (“Closest Type Strain” column) and the genomospecies to which that type strain belongs according to the 2020 genomospecies organization of the BCG (“Genomospecies” column). The genomospecies are colored differently and labeled in the “Genomospecies” column. The closest type strains are colored in different hues similar to their corresponding genomospecies color and labeled in the “Closest Type Strain” column.

The archival isolates were initially identified through traditional microbiological methods as either *B. cereus* or *B. thuringiensis* and were last classified between 1967 and 2003. Since 2003, there have been substantial increases in newly described species within the BCG, as well as proposed taxonomic changes [8,9,69,81,98]. Based on whole-genome comparison between the archival sequences and current BCG type strains using ANIb and dDDH metrics (Supplementary Table S2), we re-classified the isolates into 12 species. One isolate did not show high similarity to any BCG type strain and was classified as *Lysinibacillus capisici* in TYGS [80]. This isolate, B2797, was excluded from further analyses. The other 11 species were classified as *B. albus*, *B. anthracis*, *B. cereus*, *B. mobilis*, *B. mycoides*, *B. pacificus*, *B. paranthracis*, *B. pretiosus*, *B. cereus*/*thuringiensis*, *B. toyonensis*, and *B. tropicus* (Figure 1). Eleven archival isolates were noted as potential new species by TYGS due to their low d_4_ values across all tested type strains. These isolates were classified according to the type strain which received the highest d_4_ value and ANIb (Supplementary Table S2). The contemporary isolates were identified as *B. paranthracis* and *B. nitratireducens* (Figure 1). According to the 2020 proposed genomospecies classification scheme for the BCG [81], the isolates represent *B*. *cereus*, *s.s.*; *B. mosaicus*; *B. mycoides*; and B. *toyonensis*.

### 3.2. Pan-Genome Inference

Roary identified a total of 66,408 genes, 1647 of which were present in at least 99% of the genomes and represented the core genome of the dataset. There were 328 additional genes present in 95–99% of the genomes, 5728 in 15–95% of the genomes, and 58,705 genes in less than 15% of the genomes. The number of unique genes and the size of the pan-genome proceeded on a nearly linear positive trajectory as more genomes were added to the analysis, while the size of the core genome remained stable and close to the 1647 count from approximately the addition of the 28th genome (Supplementary Figure S1). These trends agree with previous findings suggesting the BCG has an “open” pan-genome [99].

### 3.3. Five Archival Isolates and Five Contemporary Isolates Received Annotations for at Least One Anthrax-Associated Virulence Gene

We screened all archival and contemporary isolate genome assemblies for a suite of virulence genes and virulence gene regulators important for pathogenesis in the BCG (see Section 2). Archival isolates B0818, B4510, E5429, and G9898 all received annotations for *pagA* from Prokka (Table 1, Figure 1). We explored the annotations more deeply by investigating the protein clusters identified by Roary as part of the pan-genome characterization and by aligning reference protein sequences to the isolate genomes with tBLASTn [55]. The *pagA* annotation for B0818 appeared to be spurious due to low amino acid identity between a protective antigen reference sequence (AJI08141.1) and the annotated region and being one of only two genes identified on a single, short contig (Supplementary Table S4). B4510 and E5429 had identical sequences annotated for *pagA* (Supplementary Table S4). However, these sequences were only 16% the expected length of the gene, with 380 bp compared to the expected 2294 bp (Table 1, e.g., NC_010934.1). G9898 had two annotations for *pagA*, one of which shared a protein cluster with the *B. anthracis* type strain and shared 100% amino acid identity with the protective antigen reference sequence. The second shared 61% identity. The two annotations were on separate contigs.

G9898 was the only isolate in the dataset to receive annotations for *lef* and *cya*—the toxin genes that accompany *pagA* on the pXO1 plasmid. The G9898 annotated sequences shared 100% identity with both the lethal factor (AJI08174.1) and edema factor reference sequences (AJI08175.1). This genome lacked the *capBACDE* operon but did receive annotations for two complete capsule operons (Figure 1). *bpsX-H*, which encodes an exopolysaccharide capsule, and *hasACB*, which encodes a hyaluronic acid capsule, are thought to be required for anthrax-like pathogenesis in the absence of the PGA capsule [24]. G9898 was the only isolate in this dataset with complete exopolysaccharide capsule and hyaluronic capsule operons (Figure 1).

**Figure 2:**
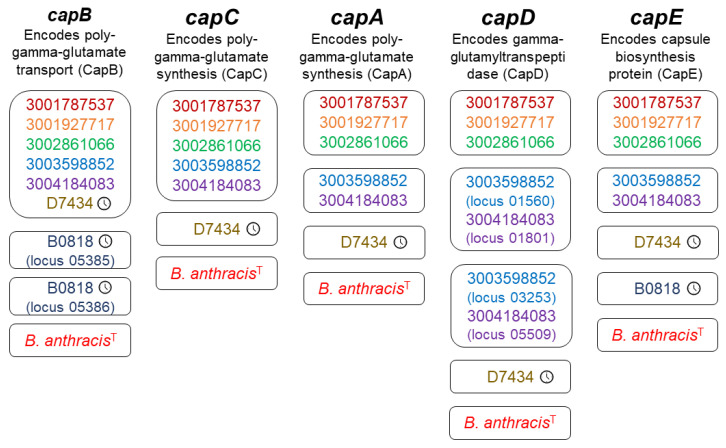
Protein clusters for each poly-y-D-glutamic acid capsule operon gene. Each box represents a protein cluster identified by Roary for each poly-y-D-glutamic acid (PGA) capsule operon gene, represented by each column of protein cluster boxes. Shared protein clusters indicate a minimum of 95% shared nucleotide identity in the annotated sequences. Each strain/isolate is represented by a different color. The clock icon to the right of the genome ID indicates an archival isolate. The “T” superscript indicates the *B. anthracis* type strain. All other genomes are contemporary isolates. Isolates that received multiple annotations for the same gene which formed different protein clusters are annotated with the locus of the annotation in the genome assembly.

Archival isolates B0818 and D7434 received two and five capsule gene annotations, respectively. B0818 received two sequential *capB* annotations (loci 05385 and 05386). Both annotated sequences shared relatively high amino acid identity with the CapB reference sequence (73.8% and 80.3%, respectively) (WP_003159943.1) but were shorter than the expected 1395 bp, with locus 05385 consisting of 389 bp and locus 05386 consisting of 623 bp (Table 1). Upon inspection of the B0818 sequences aligned with the reference sequence in MEGAX [87], it appeared that the full gene was interrupted by a stop codon. The *capE* annotation for B0818 had only 52% amino acid identity with the reference (WP_003159943.1) but was of appropriate length (140 bp compared to the 143 bp reference sequence) and was on the same contig as the *capB* annotations for this isolate (Table 1). Archival isolate D7434 received annotations for a complete capsule operon, *capBCADE*, as did all five contemporary isolates. All six isolates had *capB* sequences group together in a single protein cluster (Figure 2). D7434’s sequences had their own cluster for *capCADE* (Figure 2). The sequences for *capA* and *capD* both fell to 60% and 57% amino acid identity with the reference sequences (Table 1). This was a deviation from the other *cap* annotations in D7434 and all *cap* annotations in the contemporary isolates, which ranged from 79 to 92% (Table 1). All contemporary isolates shared a single protein cluster for *capC* (Table 1). The contemporary *B. nitratireducens* and *B. paranthracis* isolates formed separate protein clusters for *capADE* (Figure 2). The contemporary *B. paranthracis* isolates had two *capD* annotations each, which formed their own clusters (Figure 2). Upon closer inspection, *capD* was split identically in both isolates. There was no evidence of a split in the archival *B. paranthracis*, which was isolated in 1968. There were no geographic patterns reflected in these data.

### 3.4. atxA and Certhrax Annotations

In addition to the anthrax-associated virulence genes listed above, we also screened the genomes for *atxA* and *Certhrax* genes. The transcriptional activator gene *atxA* regulates virulence expression in *B. anthracis* and has been identified in atypical BCG strains that have caused anthrax-like diseases. Two distinct copies of this gene have been found in atypical BCG organisms, with the second, *atxA2*, sharing 79% nucleotide identity with *atxA1* [30]. Five genomes received at least one *atxA* annotation from Prokka (Figure 1). Of these, G9898 and B0818 also received anthrax-associated virulence gene annotations. Upon investigation at the protein level, G9898 was the only genome to share >70% amino acid identity with the AtxA1 and AtxA2 reference sequences (AJI08122.1 and AJI07941.1), with 100% amino acid identity for each gene. The other genomes shared very low amino acid identity with the reference sequences, ranging from 23 to 33% (B0818: 25.5%, D1712: 26.5%, E6797: 24% amino acid identity with AJI08122.1; B0818: 24.6%, D1712: 24.7%, E6797: 23.3% amino acid identity with AJI07941.1). The *Certhrax* gene encodes a toxin first identified in the welder’s anthrax isolate G9241 that shares structural similarity with the anthrax lethal factor [30]. Three genomes received annotations for *Certhrax*, though again, G9898 was the only one to share substantial amino acid identity with the reference sequence (G9898: 100%, F3335: 32.5%, E6442: 34% amino acid identity with EAL15945.1).

### 3.5. Four Archival Isolates and All Contemporary Isolates That Received Virulence Gene Annotations Did Not Show Evidence of Complete Bacillus Anthracis Virulence Plasmids

**Figure 3:**
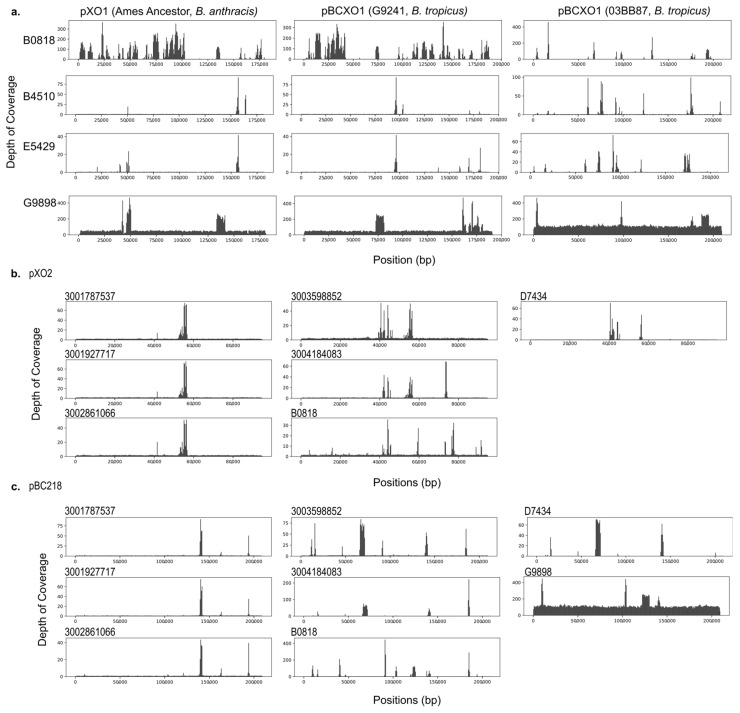
Depth of coverage for sequencing reads aligned to reference sequences. Cleaned sequence reads were aligned to reference sequences with bowtie2 to visualize the breadth (horizontal) and depth (vertical) of coverage for inferring replicon presence/absence. (**a**) pXO1 from *B. anthracis* Ames Ancestor, pBCXO1 from G9241, and pBCXO1 from 03BB87. Only G9898 (bottom row) indicates the presence of a complete pXO1 homologous plasmid. (**b**) pXO2 from *B. anthracis* Ames Ancestor. The contemporary *B. nitratireducens* (3001787537, 3001927717, and 3002861066), contemporary *B. paranthracis* (3003598852 and 3004184063), archival *B. cereus* genome B0818, and archival *B. paranthracis* genome D7434 did not indicate the presence of pXO2 despite receiving annotations for the PGA capsule operon genes. (**c**) pBC218 from G9241. In the G9241 welder’s anthrax BCG genome, the *bps* exopolysaccharide capsule genes are housed on the pBC218 plasmid. Again, G9898 was the only genome showing evidence of containing a pBC218-like plasmid.

We assessed whether there was evidence for complete or partial plasmid homologs in the isolates with virulence gene annotations by aligning the cleaned and trimmed sequencing data from each isolate to reference sequences for the plasmids of interest from *B. anthracis* and two previously characterized welder’s anthrax isolates, G9241 and 033B87. Despite full and partial virulence gene homologs being present in this dataset, only G9898 shows evidence of containing a complete *B. anthracis* plasmid homolog (Figure 2). G9898 further shows complete alignment to the pBC218 plasmid sequence from G9241 (Figure 3). The isolates containing the complete PGA capsule operon do not show evidence of having a pXO2 homolog plasmid (Figure 3). It is possible the genes are located on the chromosome or plasmid that differs dramatically from the Ames Ancestor pXO2.

### 3.6. Isolates with Virulence-Related Genes Were Distributed across the B. cereus Group

**Figure 4:**
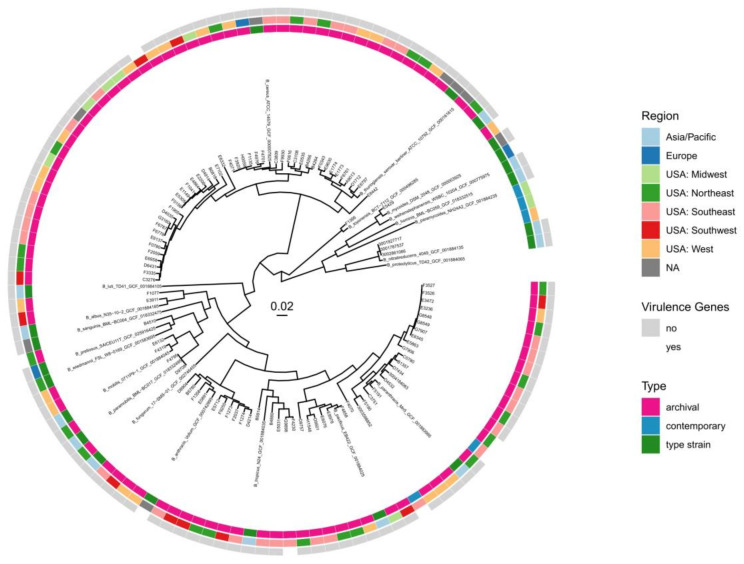
Core genome ML phylogeny. An ML phylogeny generated from the core genome alignment of 92 archival isolates, 5 contemporary isolates, and 21 BCG type strains. Three colored rings combine the genomic data with epidemiological data of each *Bacillus* isolate (n = 118). The tree is rooted with the *B. paramycoides* type strain (n = 345,224 total sites). The scale bar indicates substitutions per site. Bootstrap values were removed for ease of visualization. A tree with bootstrap values can be found in the Supplementary Information (Supplementary Figure S2).

We utilized type strain and genomospecies classification (Figure 1) with core genome ML phylogeny (Figure 4) to investigate whether there was a phylogenetic pattern in the occurrence of anthrax-associated virulence genes. The five archival isolates that received annotations for anthrax-associated virulence genes came from five different species as determined by type strain comparisons across the *B. cereus*, *s.s.*; *B. mycoides*; and *B. mosaicus* genomospecies. Notably, B4510 and E5429, which had identical truncated *pagA* sequences, came from two different genomospecies (Figure 1). The contemporary isolates came from two type species, *B. nitratireducens* and *B. paranthracis*, across two genomospecies, *B. mycoides* and *B. mosaicus*. The PGA operon genotypes of the contemporary isolates clustered together according to taxon (Supplementary Table S4). The ML phylogeny indicates the *B. nitratireducens* isolates have nearly identical core genomes, while the contemporary *B. paranthracis* isolates with virulence genes were different and distributed among archival *B. paranthracis* isolates with and without virulence gene annotations (Figure 4).

### 3.7. The Core Genome and Accessory Gene Presence/Absence Tree Topologies Are Congruent

**Figure 5:**
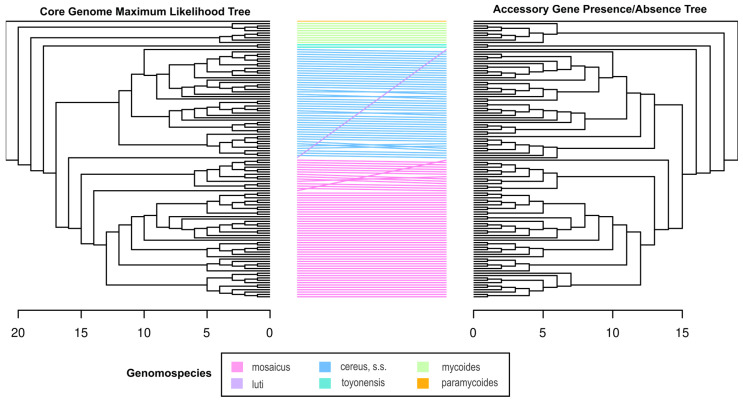
Tanglegram comparing the core genome phylogeny and accessory gene presence/absence tree. The colors of the lines connecting the tree leaves represent the genomospecies classification of each clade according to the 2020 genomospecies classification scheme for the *B. cereus* group [81] as guided by the core genome phylogeny and the distribution of type strains. The scale bars indicate the height of each tree (the largest number of edges between the tree leaves and the root). While there is entanglement within the genomospecies clades, there is no entanglement between clades, indicating the accessory gene presence/absence tree largely recapitulates the topology of the core genome phylogeny.

To determine whether the presence and absence of accessory genes across the dataset were correlated with the core genome phylogeny, we compared the binary accessory gene (presence/absence) tree topology with the core genome ML tree using a tanglegram (Figure 5). The core genome phylogeny is represented by 1647 core genes, which are shared by all isolates in the dataset. The accessory gene tree is based on the presence/absence of 64,761 accessory genes. In general, the trees had congruent topologies. There was no reorganization of leaves between the genomospecies, and notably there was no clustering of isolates in which virulence genes were detected within the accessory gene presence/absence tree. The most notable difference is the reorganization of the *B. luti* type strain between the two trees. This genomospecies is represented by a single type strain, and none of the isolates sequenced for this project clustered with *B. luti*. We quantified the strength of the correlation between the trees using Baker’s gamma index (also known as the Goodman–Kruskal–gamma index) correlation [95] and the cophenetic Spearman correlation [96]. The Baker’s gamma correlation and the cophenetic Spearman correlation were both 0.96, indicating the presence or absence of accessory genes does not supersede the core genome phylogeny.

### 3.8. There Is No Evidence of a Clock-like Signal in the Time-Structured B. cereus Group Dataset

Given the time-structured nature of the dataset, we tested whether there was a temporal signal in the core genome phylogeny by determining if there was a correlation between the phylogenetic distance from the estimated root of the tree to the leaves and ages of the leaves. Under the heuristic residual mean squared function and with the best fitting root, the R^2^ (2.9 × 10^−3^) and correlation coefficient (5.3 × 10^−2^) were extremely small, indicating there was no correlation between the isolation date and the genomic distance from the root (Supplementary Figure S3).

### 3.9. There Is a Weak Clock-like Signal in the B. cereus, Sensu Stricto and B. mosaicus Genomospecies Partitions

Despite current taxonomic ambiguity, the BCG is made up of multiple species; we chose to also assess clock-like signal within the most well-represented genomospecies in our dataset according to the most recent proposed genome-based classification scheme from Carroll and colleagues [81]: *B. cereus*, *s.s.*, and *B. mosaicus* (Figure 1). Under the heuristic residual mean squared function and using the best fitting root, the *B. cereus*, *s.s.*, partition had an R^2^ of 0.019 and correlation coefficient of 0.138 (Supplementary Figure S3). The *B. mosaicus* partition had a slightly stronger clock-like signal with an R^2^ of 0.083 and correlation coefficient of 0.288 (Supplementary Figure S3). Though weak, these statistics indicate an association between genomic distance from the root and the isolation date in contrast to the whole BCG.

## 4. Discussion

Here, we produced 98 genomes from archival and contemporary isolates collected between 1967 and 2023, 97 of which were confirmed to be from the BCG. In 10 isolates, we identified DNA sequences homologous to virulence genes found in classically defined *B. anthracis*. Though these genomes were spread throughout the BCG phylogeny, the older isolates with homologous sequences showed evidence of pseudogenization and gene decay. Our pan-genome analysis indicated no meaningful sharing of accessory genes between lineages in the BCG. Though the temporal signals we identified in the BCG, *B. cereus*, *s.s.*, genomospecies, and *B. mosaicus* genomospecies were weak or absent, there were many factors aside from recombination that could have confounded the clock signal. Ultimately, we found no strong evidence of the virulence genes (intact or otherwise) being distributed throughout the BCG by gene transfer.

The most striking genome assessed in this study, G9898, was the only isolate analyzed that possessed anthrax toxin genes and full exopolysaccharide and hyaluronic acid capsule operons. This isolate was derived from a case of fatal pneumonia in a Louisiana welder in 1996 [100], and was previously shown to express a capsule [101]. As the only *B. tropicus* isolate in the dataset with anthrax-associated virulence genes, it fits into the growing set of welder’s anthrax isolates identified as *B. tropicus* [18,23].

The *pagA* sequences identified in B4510/E5429 and the *capB* sequences identified in B0818 were likely non-functional due to their interruption by stop-codons. Though the *cap* operon annotated in D7434 was complete, the amino acid identity for *capACD* compared to the reference sequences was below 80%, suggesting the capsule may have differed phenotypically for this strain. Additionally, the *capD* sequences from the contemporary *B. paranthracis* isolates appear to be interrupted in a similar fashion to the *capB* sequence in B0818. These findings may indicate a larger pattern of anthrax-associated virulence gene pseudogenization.

Though plasmid transfer has been demonstrated between different lineages of the BCG in experimental and environmental contexts through transduction and conjugation [8], we did not see evidence in this dataset of natural, large-scale gene exchange between lineages. We only see evidence of a full pXO1 homolog plasmid in G9898, the welder’s anthrax case. The remainder of the isolates, including the contemporary isolates encoding the full *cap* operon, did not show evidence of possessing complete homolog plasmids. The genes and gene fragments identified here are either on the chromosome or a different plasmid. This could be determined definitively in the future through long-read sequencing. We furthermore found that while we do see anthrax-associated virulence genes spread throughout different lineages of the BCG, the presence and absence of accessory genes largely recapitulates the topology of the core genome phylogeny. This would suggest there is not frequent gene transfer between the BCG genomospecies lineages represented in the dataset such that they would meaningfully override the core genome phylogeny. This study recapitulates previous findings in support of the linear inheritance of anthrax-associated virulence genes rather than acquisition by gene transfer, particularly by plasmid transfer [94].

However, one line of evidence presented here indicates gene transfer occurs in the BCG in general. Despite the congruence between the core genome and accessory gene presence/absence trees, the pan-genome analysis did recapitulate previous findings suggesting that the BCG pan-genome is open [98] and members of the BCG are regularly acquiring new and unique genes (Supplementary Figure S1). The most likely source of this observed gene diversity is gene transfer [102,103].

The lack of a temporal signal in the whole BCG core genome SNP-based ML phylogeny with estimated isolation dates spanning 1872–2023 (Supplementary Tables S1 and S3) could arguably indicate gene transfer. However, we found weak temporal signals when the BCG was separated into genomospecies partitions for *B. cereus*, *s.s.*, and *B. mosaicus*. Recombination and hypermutation are the two nucleotide-level biological processes known to disrupt strict clock-like patterns in phylogenies due to variant mixing between lineages [97]. It is also possible the BCG life history and lifestyle of sporulation and intermittent dormancy contributes to the lack of a clock-like signal due to extended and/or irregular generation times [104]. Different populations within and between lineages may enter dormancy for varying amounts of time and reproduce at different rates, contributing to a seemingly stochastic phylogenetic signal.

Our findings regarding the apparent pseudogenization of anthrax-associated virulence gene homologs suggest they were not acquired by any lineage in which they are present in recent history. Rather, they may be an illustration of gene deletion in progress, which acts as a balance to the open pan-genome for maintaining a reasonable genome size [105]. When we consider the pseudogenization of the anthrax-associated virulence genes together with the lack of evidence for transfer between lineages by means of gene transfer, the most likely explanation for their presence throughout the BCG is linear inheritance from a common ancestor. While the genes featured here are crucial for the pathogenesis of anthrax disease, most BCG bacteria are not obligate pathogens and must survive and compete in diverse ecological niches [8,69,70]. It is plausible that most descendant lineages of this common ancestor, possessing the complete set of toxin and PGA capsule genes, diversified into niches where the virulence genes were not advantageous. While the virulence genes may not have been deleterious, they did not confer a selective advantage. Mutations or deletions may have accumulated in them through genetic drift, causing their pseudogenization and eventual loss in most of the BCG. In contrast to the rest of the BCG, the highly conserved pathogenic *B. anthracis* depends on infecting a mammalian host to emerge from its spore state and reproduce [94], and has a “closed” pan-genome, meaning the number of unique genes added to the *B. anthracis* pan-genome with each genome added decreases to zero [106]. The phenomenon of genomic reduction by pseudogenization and reduced gene transfer on the evolutionary path from environmental microbe/opportunistic pathogen to obligate pathogen has been well described [107,108,109], and gene loss as a signal of ecological diversification in bacteria has been shown recently among *Ruegeria* [110] and *Bordetella* [111].

This study and the time-structured set of BCG isolates have some limitations and offer opportunities for several further analyses that were beyond the scope of this manuscript. While we leveraged whole-genome sequencing data and a pan-genome approach to investigate the possibility of gene transfer within the BCG regarding specific virulence genes, we did not pursue a formal inference of recombination within this dataset. A formal inference of recombination may be fruitful to explore using subsets of the BCG data presented here in the future. This study also depended on whole-genome short-read sequencing data. Supplementing the short-read data with long-read data would help resolve the genomic structure(s) found in each isolate and the locations of the anthrax-associated virulence genes we identified. We were also unable to leverage detailed clinical data to understand the disease/infection from which each isolate was derived due to a lack of detailed records. Detailed information on clinical presentation, as well as laboratory-based phenotypic analyses, such as capsule expression and characterization, would also contribute to our knowledge of how anthrax-associated virulence genes, when present, are being expressed. Phenotypic analyses of a selection of the isolates sequenced here will be presented in future studies. As we identified ten BCG genomes by whole-genome nucleotide identity (dDDH and ANI) as *B. anthracis* that lacked any pXO1 or pXO2 virulence genes or pseudogenes, it would be fruitful to investigate the gene content of these isolates and their phylogenetic relationships to previously characterized virulent *B. anthracis* strains. Lastly, this dataset is not representative of the whole diversity of the BCG. While we do not find evidence of horizontal gene transfer in the bacterial populations represented in this dataset, we cannot rule out this mechanism of virulence gene acquisition across all populations in the BCG. It is possible the scenario of how anthrax-associated virulence spread across the known BCG is more complex than that presented here. The continued characterization of environmental and clinical BCG isolates is crucial to further enhance our understanding of virulence in the complex.

In this study, we share evidence for homology to and fragmentation of anthrax-associated virulence genes across multiple species and/or genomospecies of the BCG that cannot be classified as *B. anthracis*. This study, along with a growing body of clinical case reports and microbiological research, supports the etiology of anthrax disease being toxin-mediated, rather than being a narrowly taxonomically defined bacterium.

## Figures and Tables

**Table 1:** Isolates with annotations for pXO1 and pXO2 anthrax-associated virulence genes. Entries in the “Gene Length (bp)” column are bolded for annotations that were truncated compared to the reference sequence gene length.

	Isolate Name	Isolation Source	Isolation Location (Country: State)	Isolation Year	Closest Species Type Strain	Gene	Amino Acid Identity (%)	Gene Length (bp)	Reference Gene Length (bp)
Archival	B0818	Pustule	USA: NM	1968	*B. cereus*	pagA *	32.35	**1628**	2294
						*capB_1*	80.33	**389**	1395
						*capB_2*	73.81	**623**	1395
						*capE*	52.4	140	143
	B4510	Ulcer	USA: MA	1970	*B. pretiosus*	*pagA*	34.71	**380**	2294
	D7434	Blood	USA: NY	1976	*B. paranthracis*	*capB*	90.52	1130	1395
						*capC*	84.5	449	449
						*capA*	60.26	1199	1235
						*capD*	57.17	1615	1587
						*capE*	57.14	143	143
	E5429	Blood	USA: CO	1979	*B. mycoides*	*pagA*	34.71	**380**	2294
	G9898	Sputum	USA: LA	1996	*B. tropicus* *†*	*pagA_1*	100	2294	2294
						*pagA_2*	61.31	2282	2294
						*lef*	100	2429	2429
						*cya*	100	2402	2402
Contemporary	3001787537	sputum	USA: IL	2019	*B. nitratireducens*	*capB*	89.88	1181	1395
						*capC*	91.95	449	449
						*capA*	87.08	1211	1235
						*capD*	82.52	1586	1587
						*capE*	82.6	143	143
	3001927717	urine	USA: ND	2020	*B. nitratireducens*	*capB*	89.88	1181	1395
						*capC*	91.95	449	449
						*capA*	87.08	1211	1235
						*capD*	82.52	1586	1587
						*capE*	82.6	143	143
	3002861066	urine	USA: CA	2022	*B. nitratireducens*	*capB*	89.88	1190	1395
						*capC*	91.95	449	449
						*capA*	87.08	1211	1235
						*capD*	82.52	1586	1587
						*capE*	82.6	143	143
	3003598852	blood	USA: TN	2022	*B. paranthracis*	*capB*	90.15	1130	1395
						*capC*	92.62	449	449
						*capA*	87.86	1255	1235
						*capD_1*	79.69	**1043**	1587
						*capD_2*	83.3	**431**	1587
						*capE*	84.78	143	143
	3004184083	blood	USA: AR	2023	*B. paranthracis*	*capB*	90.15	1130	1395
						*capC*	91.95	449	449
						*capA*	87.86	1255	1235
						*capD_1*	79.69	**1043**	1587
						*capD_2*	83.3	**431**	1587
						*capE*	84.78	143	143

* The *pagA* annotation for B0818 was likely spurious; *†* G9898 was noted as a potential new species by TYGS due to its low dDDH values with all BCG type strains. However, G9898 had the highest similarity by ANI and dDDH values to the *B. tropicus* type strain.

## Data Availability

The 16S rRNA reference sequences came from NCBI BioProjects PRJNA33175 and PRJNA33317. Virulence gene reference sequence accessions are in Supplementary Table S3. Type strain genome and BioSample accessions are in Supplementary Table S1. Plasmid reference sequences can be found under the following accessions: pXO1 from the Ames Ancestor *B. anthracis* (NC_007322.2), pBCXO1 from the welder’s anthrax isolate G9241 (NZ_CP009592.1), pBCXO1 from the welder’s anthrax isolate 033B87 (NZ_CP009940.1), pXO2 from the Ames Ancestor *B. anthracis* (NC_007323.3), and pBC218 from G9241 (NZ_CP009591.1). The genome sequence data produced in this study are available in NCBI BioProject PRJNA1122551.

## Supplementary Materials

The following supporting information can be downloaded at https://www.mdpi.com/article/10.3390/pathogens13100884/s1, Figure S1: Pan-genome plots from Roary; Figure S2: Maximum likelihood tree with bootstrapping scores; Figure S3: Tip-to-root distance vs. time plot; Table S1: Reference type strain information; Table S2: All isolates with metadata, including year, location, and sequencing information (coverage, dDDH, ANIb, GC content, N50, and # of contigs); Table S3: Custom virulence gene database for Prokka and BLAST; Table S4: Protein clusters.
