# Supplementary material for: Investigating Anthrax-Associated Virulence Genes among Archival and Contemporary Bacillus cereus Group Genomes
Source: Pathogens. 2024 Oct 10;13(10):884. doi: 10.3390/pathogens13100884 (PMC11510535; doi:10.3390/pathogens13100884)
Supplement: Supplementary file 1 [file pathogens-13-00884-s001.zip › Sabin et al Supplementary Information.pdf]

Supplementary Information

For "Investigating Anthrax-Associated Virulence Genes Among Archival and Contemporary *Bacillus cereus* Group Genomes"

Supplementary Figures

a.

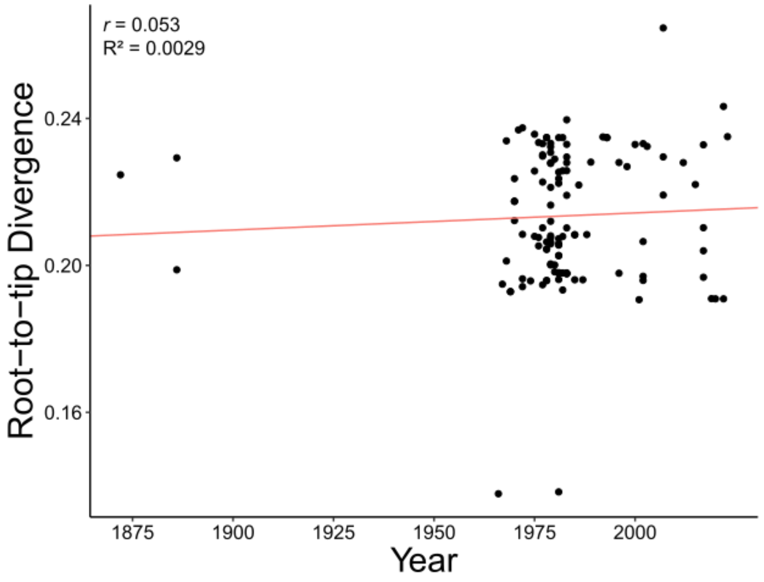

b.

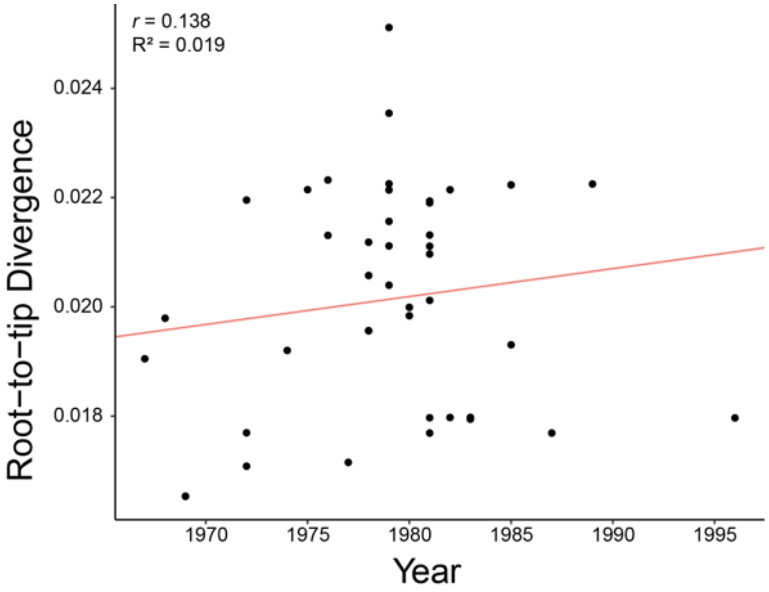

c.

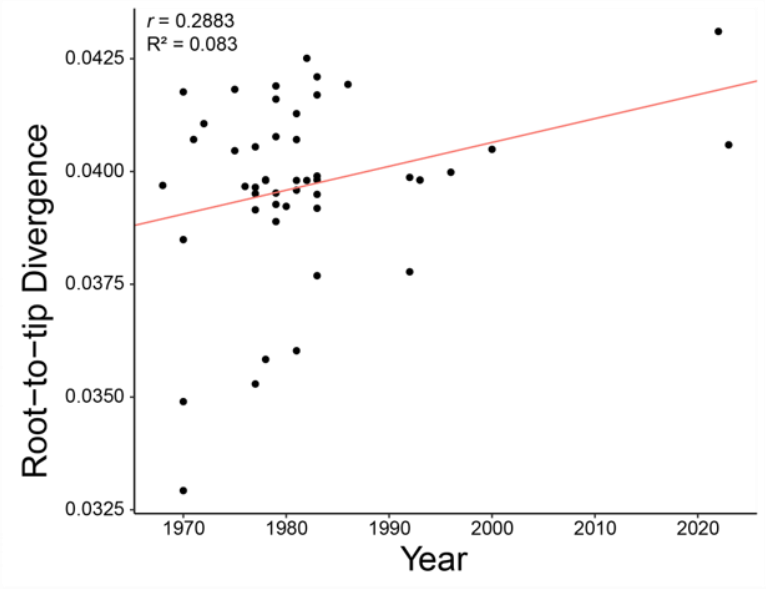

Supplementary Figure 1. Clock-like signal test plots. The root-to-tip divergence between the best fitting root and each genome plotted against the inferred year of isolation. Each point represents one genome. The correlation coefficient (*r*) and R^2 are noted at the top left of each plot. Divergence and correlation statistics were calculated in TempEst (Rambaut et al., 2016). Plots shown represent the *Bacillus cereus* group dataset (**a**), the *Bacillus cereus, s.s.* genomospecies dataset (**b**), and the *Bacillus mosaicus* genomospecies dataset (**c**).

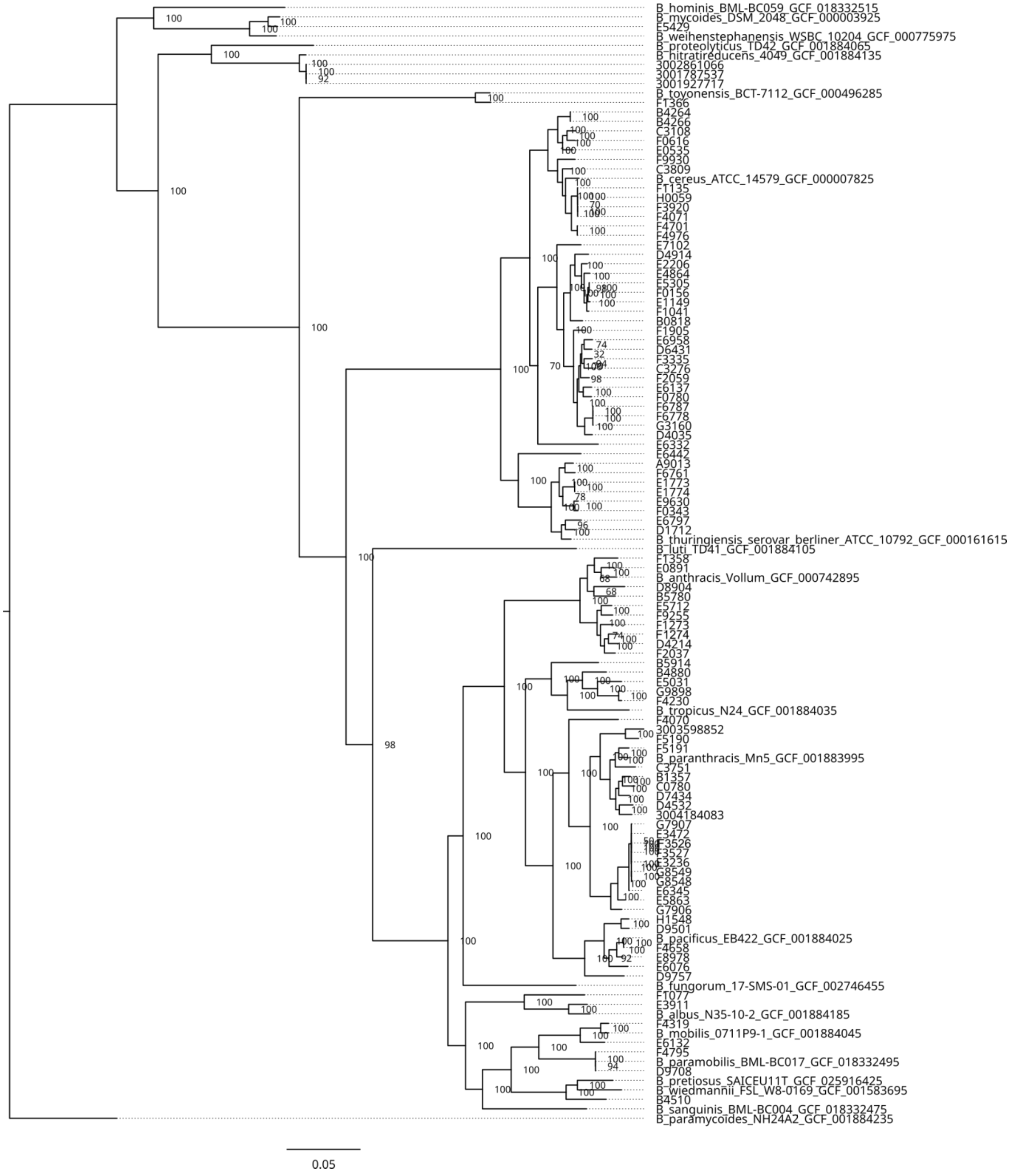

Supplementary Figure 2. Maximum likelihood tree with bootstrap values. This is the same phylogeny depicted in Figure 3 of the main text with bootstrap values included at each node. The scale bar indicates substitutions per site.

a.

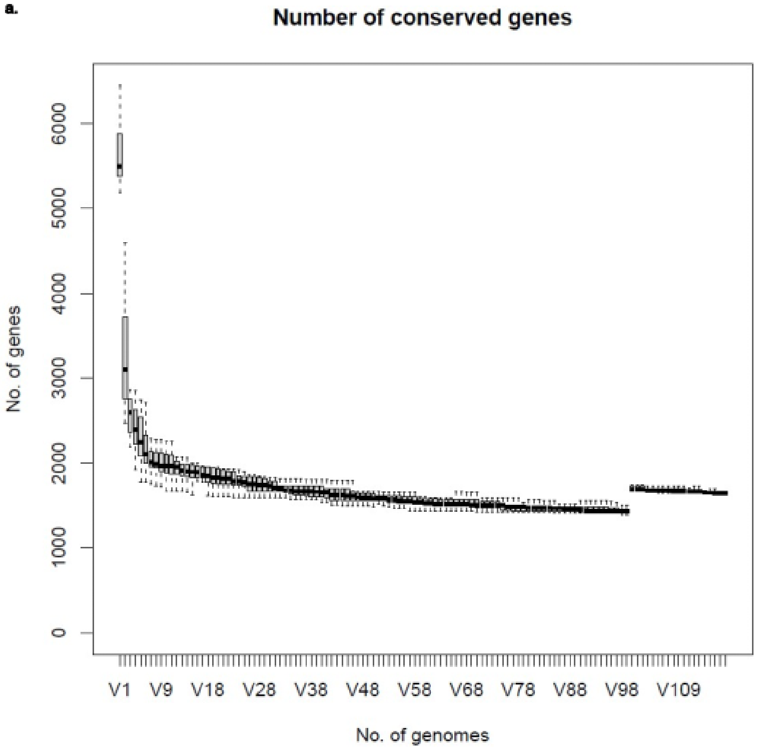
Number of conserved genes
No. of genes
0
1000
2000
3000
4000
5000
6000
V1
V9
V18
V28
V38
V48
V58
V68
V78
V88
V98
V109
No. of genomes

b.

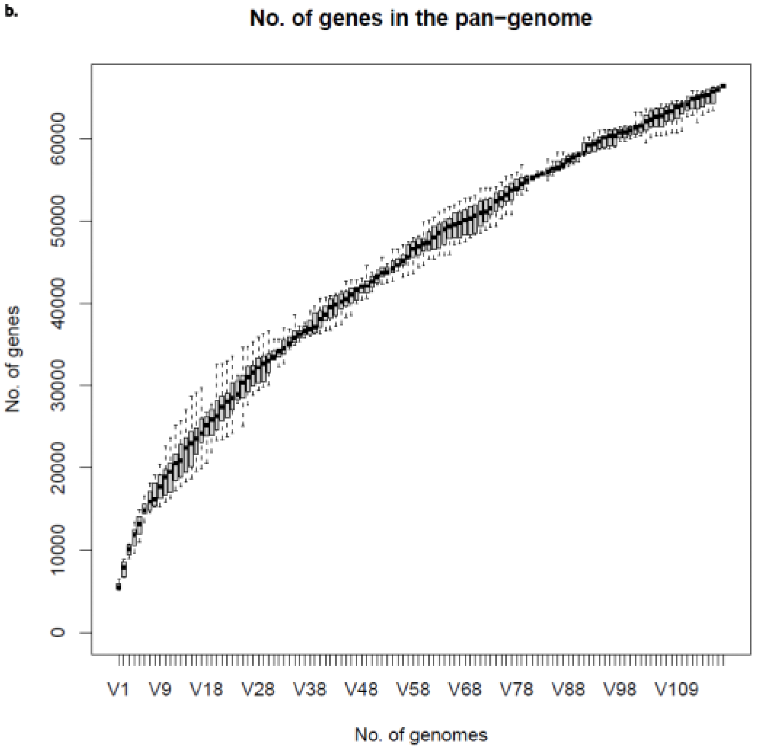
No. of genes in the pan−genome
No. of genes
0
10000
20000
30000
40000
50000
60000
V1
V9
V18
V28
V38
V48
V58
V68
V78
V88
V98
V109
No. of genomes

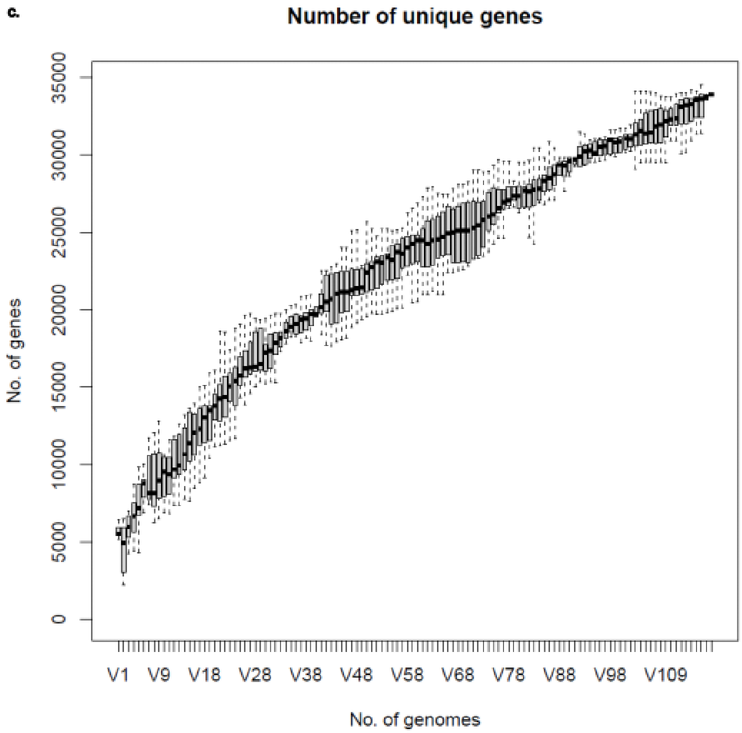

Supplementary Figure 3. *Bacillus cereus* group pan-genome plots from Roary. The number of conserved genes (**a**), total genes (**b**), and unique genes (**c**) in the pan-genome with the addition of each new genome. The number of conserved genes plateaus and remains relatively stable as more genomes are added to the pan-genome dataset. The number of total genes and unique genes continues to increase with the addition of each genome to the dataset.

Supplementary Tables

All supplementary tables can be found in the file supplementary_tables.xlsx.

Supplementary Table 1. Type strain metadata

Type strain names, GenBank accessions, BioSample accessions, inferred original specimen collection date, primary type strain genome references, and whether the type strain was included in the core genome and subsequent analyses.

Supplementary Table 2. Isolate metadata

Metadata and genome information for all archival and experimental isolates including year received, source information (original specimen type and/or associated disease as available), geographical origin, type strain genome server (TYGS) potential new species (yes/no), TYGS top species according to digital DNA:DNA hybridization (dDDH) comparison to bacterial type strains, TYGS dDDH d_4 statistic for best match type strain, TYGS dDDH lower boundary of confidence interval for d_4 statistic, TYGS dDDH upper boundary of confidence interval for d_4 statistic, average nucleotide identity (ANI) best matched type strain among *Bacillus cereus* group type strains, bidirectional ANI (ANIb) statistic for best match, ANIb statistic standard deviation, genomic assembly mean depth of coverage (with down-sampling prior to assembly), checkM2 general completeness, checkM2 contamination estimate, checkM2 specific

completeness, coding density, N50, average gene length, genome size, GC content, and total coding sequences.

Supplementary Table 3. Custom virulence gene database for Prokka and BLAST

Locus tag, accession, gene symbol, and gene description for each gene included in the custom amino acid sequence file used as a custom database for Prokka and BLAST.

Supplementary Table 4. Pan-genome protein clusters

A table of protein clusters identified in the Roary pan-genome analysis.
